# MiR-101-3p Promotes Tumor Cell Proliferation and Migration via the Wnt Signal Pathway in MNNG-Induced Esophageal Squamous Cell Carcinoma

**DOI:** 10.3390/toxics12110824

**Published:** 2024-11-18

**Authors:** Jianding Wang, Wenwen Zhang, Rui Zhang, Hanteng Yang, Yitong Li, Junling Wang, Chengyun Li

**Affiliations:** 1 Department of Toxicology, School of Public Health, Lanzhou University, Lanzhou 730000, China; 220220912851@lzu.edu.cn (J.W.); zww19950101@163.com (W.Z.); liyt2018@lzu.edu.cn (Y.L.); wangjl@lzu.edu.cn (J.W.); 2 Key Laboratory for Reproductive Medicine and Embryo, The Reproductive Medicine Special Hospital of the Lanzhou University First Affiliated Hospital, Lanzhou 730000, China; guairain_1125@hotmail.com; 3 Department of General Surgery, Lanzhou University Second Hospital, Lanzhou 730000, China; yanghanteng@126.com

**Keywords:** MNNG, miR-101-3p, esophageal squamous cell carcinoma, regulatory mechanisms, biomarker

## Abstract

N-methyl-n’-nitroso-n’-nitroso guanidine (MNNG) can induce esophageal squamous cell carcinoma (ESCC), and microRNAs are associated with the development of ESCC and may serve as potential tumor prognostic markers. Thus, the aim of this study was to evaluate the potential function of miR-101-3p in MNNG-induced ESCC. An investigation of risk factors in patients with ESCC was carried out and the concentration of nine nitrosamines in urine samples was detected by the SPE-GC-MS technique. Then, we performed cancer tissue gene sequencing analysis, and RT-qPCR verified the expression level of miR-101-3p. Subsequently, the relationship between miR-101-3p potential target genes and the ESCC patients’ prognosis was predicted. Finally, we investigated the function of miR-101-3p in MNNG-induced ESCC pathogenesis and the regulatory mechanism of the signaling pathway by in vivo and in vitro experiments. The results revealed that high dietary nitrosamine levels are high-risk factors for ESCC. MiR-101-3p is down-regulated in ESCC tissues and cells, and its potential target genes are enriched in cell migration and cancer-related pathways. MiR-101-3p target genes include AXIN1, CK1, and GSK3, which are involved in the regulation of the Wnt signaling pathway. MiR-101-3p overexpression promotes apoptosis and inhibits the proliferation and migration of Eca109 cells. The Wnt pathway is activated after subchronic exposure to MNNG, and the Wnt pathway is inhibited by the overexpression of miR-101-3p in Eca109 cells. Down-regulated miR-101-3p may exert tumor suppressive effects by regulating the Wnt pathway and may be a useful biomarker for predicting ESCC progression.

## 1. Introduction

Esophageal cancer (EC) is the primary malignant tumor of the esophageal mucosal epithelium, and a serious threat to the health of people around the world. Each year, there are about 330,000 new cases of EC and more than 270,000 EC-related deaths reported worldwide, with more than half of them occurring in China. Esophageal squamous cell carcinoma (ESCC) is the major pathological type of EC and accounts for more than 90% [1]. Epidemiological studies have confirmed that ESCC risk factors include heavy drinking, smoking, betel chewing, nutritional deficiencies, and nitrosamines [2]. Despite major improvements in diagnostic and therapeutic approaches to ESCC, 5-year survival rates for patients with advanced ESCC remain poor [3]. Meanwhile, effective diagnostic and prognostic biomarkers of ESCC are still lacking. Therefore, the improvement of diagnostic efficiency for ESCC, the validation and identification of diagnostic biomarkers, and the exploration of the molecular mechanisms of ESCC have become the focus of recent public health research.

A study of the Huai’an area found that ESCC patients had significantly higher levels of nitrite exposure than the healthy population, N-methyl-n’-nitroso-n’-nitroso guanidine (MNNG) can induce the malignant transformation of normal cells of esophageal mucosa Het-1A [4]. Preserved foods are high in MNNG, and, in recent years, studies have examined the correlation between diets high in MNNG and ESCC, but the specific regulatory mechanisms are unclear [5]. It has been reported that microRNAs (miRNAs) with 18–23 nucleotides play important roles in regulating the progression of many diseases [6,7]. Evidence also suggests that miRNA can play crucial roles in the occurrence and developmental progress of human cancers [8]. Some miRNAs have been identified as participants in the regulation of cancer pathogenesis, including the proliferative, apoptosis, invasiveness, and cycle of cancer cells, etc. [9]. Therefore, understanding the function of miRNAs in ESCC tumorigenesis development is crucial.

In this study, we investigated the risk factors for the high incidence of ESCC in the Wuwei area; then, we detected the actual expression levels of key miRNA in the ESCC tissue samples. Moreover, we systematically analyzed the relative expression levels of key miRNAs in ESCC tissues from The Cancer Genome Atlas (TCGA) and Gene Expression Omnibus (GEO) databases. In addition, we also detected the actual expression levels of key miRNA in the tissue samples of 32 newly diagnosed ESCC patients and the ESCC cell line by RT-qPCR. Subsequently, we further investigated the potential regulatory target genes and their relationship to the prognosis of ESCC patients using bioinformatic analyses. Finally, we further analyzed the biological functions of key miRNA in vitro and investigated the regulatory mechanisms in ESCC development.

## 2. Materials and Methods

### 2.1. Epidemiological Survey and Specimen Collection

To understand the risk factors of ESCC in high incidence of ESCC in Wuwei area of Gansu Province, this study investigated the basic situation and lifestyle of 60 patients with ESCC in Wuwei area (ESCC average annual incidence 45.23/100,000) and 180 normal control groups in Jingtai (ESCC average annual incidence 9.83/100,000). The contents of the investigation mainly include the basic information of the subjects and the relevant information such as living and eating habits. In addition, 32 ESCC patients were recruited from Wuwei Tumor Hospital in Gansu Province, including 27 males and 5 females, and clinical epidemiological investigations were carried out on each study subject. Ten cases were randomly selected as the experimental group from the above case group, and healthy control group was selected from areas with a low incidence of ESCC, who did not suffer from any digestive system disease and were matched by gender and age. Morning urine was collected from the ESCC patients and the control group, 0.1–0.5 g of NaOH was added as a preservative, and the urine was transported back to the laboratory within 12 h at 10 °C and stored at −80 °C for measurement. According to Declaration of Helsinki, this study was approved by the Ethics Committee of School of Public Health, Lanzhou University (No: LZUGWLL-180301) and obtained informed consent from patients.

### 2.2. Nitrite Detection in Urine Samples and RNA Sequencing in Tissue Samples

The concentration of nine N-nitrosamines in urine specimens was determined by solid phase extraction (SPE) coupled with gas chromatography–mass spectrometry (GC-MS). The concentration of 8 N-nitrosamines in urine was determined by SPE-GC-MS. Take 5 mL of urine and re-melt it in a 37 °C water bath. Extract and elute it according to the instructions. Extraction, elution, nitrogen blowing, and on-line detection were carried out according to the instructions.

ESCC patients’ tumor tissues and paraneoplastic tissue specimens of three newly developed ESCCs among permanent residents in Wuwei City, Gansu Province were selected for gene microarray high-throughput miRNA detection (Illumina Array 1 × 90K microRNA microarray high-throughput detection). The high-throughput sequencing data of the chip can be downloaded from the public website platform https://figshare.com/articles/dataset/Chengyun_Li-sequencing_test_report--miRNA_zip/25913128 (accessed on 28 May 2024).

### 2.3. MiR-101-3p Expression Validation in the TCGA, GEO Genome Databases, ESCC Patients Tissue Samples, and Cell Line

We collected ESCC cases from the TCGA database using the R/BioConductor software 4.4.1 package ‘TCGAbiolinks’ tools (up to 1 October 2022). The TCGAbiolinks package provides multiple methods for analysis, including the standardization of data from different sequencing sources and differential expression analysis. This study was conducted in compliance with the publication guidelines of TCGA portal platform http://cancergenome.nih.gov/publications/publicationguidelines (accessed on 10 February 2022). Then, we checked all these downloaded datasets and excluded the samples without matched RNA sequencing data, samples of ESCC patients who had received radiotherapy or chemotherapy treatment, and samples of patients with other tumors besides ESCC. Finally, 172 samples were included (161 ESCC tumor samples and 11 adjacent samples for non-tumor samples).

To enhance the comparability and reliability of our detection and TCGA analysis results, we collected RNA sequencing expression datasets from the cancer tissues of 119 ESCC patients and adjacent normal esophageal tissues from the GEO database. The tissue samples of the 119 ESCC patients and the RNA sequencing datasets from GEO were downloaded, and the original RNA sequencing data were normalized using the Voom function and trimmed mean of M-values (TMM) normalization methods. The Voom function was applied with the highest degree of discreteness to the RNA-seq data after normalizing the chip expression data into an expression matrix, and then the t-test was used for the analysis. According to the stated requirements, one dataset was included, with accession number GSE43732. Finally, we verified and comprehensively analyzed the miR-101-3p relative expression levels in GSE43732 ESCC tissue samples.

The expression levels of miR-101-3p in the tissue samples of 32 ESCC patients, the ESCC human Eca109 cell line, and human esophageal epithelial cell (HEEC) lines were tested and analyzed by RT-qPCR. RNA was isolated from tissues and cells by using the TRIzol (Invitrogen, CA, USA). Reverse transcription and PCR test was performed using the Prime Script™ RT kit with gDNA eraser (Takara, Japan), TB Green Premix Ex Tap II (Takara, Japan), and U6 as the endogenous control. The miR-101-3p and U6 sense primer are shown in Table 1.

### 2.4. MiR-101-3p Target Genes Prediction and Functional Analysis

According to the complementary matching principle of base pairs, target genes were predicted using the miRanda, miRWalk, starBase, PITA, CoMeTa, RNA22, and TargetScan online analysis tools. The genes predicted in at least three tools were chosen as the miR-101-3p target genes for further analysis. The involved function annotation status and potential signaling pathway regulation of these genes were then analyzed using the DAVID and KOBAS 3.0 online bioinformatics analysis tools. Finally, the core genes were selected by the protein–protein interaction (PPI) networks based on connectivity scores using the Cytoscape v3.6 software.

### 2.5. Esophageal Injury Rats Modeled by Subchronic Exposure to MNNG and Hematoxylin-Eosin Staining

A total of 75 Wistar rats were randomly divided into five groups, including 0.08 mg/kg, 0.12 mg/kg, 0.16 mg/kg, and 0.20 mg/kg MNNG groups. The rats underwent continuous MNNG intragastric poisoning 5 days a week, and, at the same time, they were given 30 μg/mL MNNG ad libitum for thirteen weeks to construct an animal model of esophageal precancerous lesions in rats. Control group rats were gavaged with saline and drank ultrapure water. The tissues were subjected to a series of processing steps, including paraffin embedding, deparaffinization, hydration, hematoxylin staining, destaining with acidic water and ammonia, dehydration using a gradient alcohol solution, eosin staining, and sealing. Subsequently, we observed it under the microscope and scored it by a combination of the degree of inflammatory cell infiltration, epithelial cell necrosis, and peritubular inflammatory cell infiltration, with the absence of foci regarded as negative and scored as 1, mild as 2, moderate as 3, and severe as 4. Study was approved by the Ethics Committee of School of Public Health, Lanzhou University (No: LZUGWLL-180301).

### 2.6. Cell Culture, Cell Staining and Cell Transfection

We obtained the Eca109 and HEEC lines from Hunan Platzer Biotechnology Co., Ltd. (Changsha, China). We cultured all the cell lines in DMEM (GE Health Care HyClone™, Logan, UT, USA) medium and supplemented them with 10% fetal bovine serum (FBS), 100 U/mL penicillin, and 100 mg/mL streptomycin in 37 °C with 5% CO_2_.

Eca109 cells in logarithmic growth phase were inoculated into 6-well plates at 3 × 10^5^, and after wall attachment, the cells were stained with 0 μg/mL, 3 μg/mL, and 5 μg/mL of MNNG for 48 h according to the groups.

We then inoculated the Eca109 cells (3 × 10^5^) into a 6-well plate and placed the cells that showed a cell growth of 50–70% in a medium without penicillin–streptomycin solution for transfection. The miR-101-3p mimic and negative control transfection reagents were synthesized by RiboBio (Guangzhou, China). We used negative control transfection as a negative control group, and the transfected cells were used as a blank control group. We then used RT-qPCR to test miR-101-3p mimic expression and evaluate the transfection efficiency.

### 2.7. In Vitro Cell Proliferation, Migration Cell Cycle and Apoptosis Assay

For the logarithmic growth phase, we inoculated Eca109 cells and mimic-transfected cells in a 96-well plate, with 5 × 10^3^ cells per well, and set up five replicate wells in each group. The CCK8 method was used to evaluate the effect of cell proliferation after transfection. The microplate reader then detected the OD value, analyzed the values, and drew a growth curve.

Then, inoculated logarithmic growth phase Eca109 cells and mimic-transfected cells in a 6-well plate, with 5 × 10^5^ cells per well; 48 h after transfection, we scratched the cells with a 200 µL pipette tip, washed them three times with PBS, and added the complete medium for culture. We took pictures and recorded the scratches at 0 h, 24 h, 48 h, 72 h, and used Image pro plus to analyze cell migration ability.

For the cell cycle and apoptosis analysis, we harvested the Eca109 cells and transfected the Eca109 cells. For the cell cycle analysis, cell pellets were added with RNAase for 30 min at 37 °C, then stained with propidium iodide (PI) and incubated, avoiding light, for 30 min at 4 °C. Cell apoptosis was detected using the Annexin V-APC-7AAD apoptosis kit (MultiSciences Biotech, Hangzhou, China). The detection of apoptosis was quantified by the FACS Calibur Flow cytometer (BD American, Franklin Lakes, NJ, USA).

### 2.8. Western Blotting Assay and Immunohistochemistry

SDS-PAGE was used to separate protein bands and transfer them to the nitrocellulose membrane. Add 10 µL of sample per well, perform electrophoresis at a constant voltage of 80 V for 40 min, and switch to constant voltage of 120 V until the end. Then, a constant current of 220 mA was applied to the PVDF membrane for a duration of the protein’s molecular weight plus 30 min. Next, use 5% skimmed milk to block the membrane for 2 h, and then use 5% bovine serum albumin to incubate primary antibodies AXIN1 (Rabbit mAb No.#3323), Ck1 (Rabbit mAb No.#2655), β-Catenin (Rabbit mAb No.#8480), p-β-Catenin (Rabbit mAb No.#4176), TCF7 (Rabbit mAb No.#2206), GSK-3α (Rabbit mAb No.#4818), and p-GSK-3α (Rabbit mAb No.#9327) (Cell Signaling Technology, Danvers, MA, USA) at 4 °C for 12 h. We then used corresponding secondary antibodies (Boster Biological Technology, Pleasanton, CA, USA. Goat Anti-Rabbit IgG, No. BA1054) to incubate for 1 h. Finally, we used the general ECL chemiluminescence imaging kit (Thermo Fisher Scientific, Waltham, MA, USA) to visualize the protein band signals. Finally, we used immunohistochemical methods to observe the actual expression levels of some key proteins in the wnt pathway in the esophageal tissues of rats with subchronic exposure to MNNG.

### 2.9. Statistical Analysis

All biological experiments were conducted in triplicate. Continuous variables are expressed as means and their corresponding standard deviations. Analysis of variance (ANOVA), Chi-square test, or non-parametric tests were employed as appropriate for data comparisons. The data analysis was conducted using IBM SPSS Statistics 26.0 (SPSS Inc., Chicago, IL, USA) and GraphPad Prism 8.0 (La Jolla, CA, USA). The threshold for statistical significance was set at *p* < 0.05.

## 3. Results

### 3.1. Association Between the Diet Lifestyle Behaviors and Risks of ESCC

The statistical analyses of risk factors for ESCC showed a higher prevalence of ESCC in people who drank tea more than 5 times per week, and the prevalence of ESCC is higher among individuals who consume well, lake, or river water in comparison to those who consume tap water. The incidence of ESCC was markedly elevated in subjects who habitually consumed preserved foods (≥4 days/week) in comparison to those who ingested minimal (1–3 days/week) or no amounts of such foodstuffs (Table 2).

**Figure 1:**
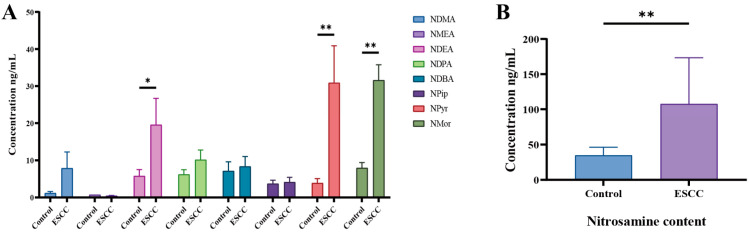
Association between the diet lifestyle behaviors and risks of ESCC. (**A**) Levels of nine N-nitrosamines (NDMA, NMEA, NDEA, NDPA, NDBA, Npip, Npyr, and Nmor) in urine samples of ESCC patients and healthy controls. (**B**) Total nitrosamines in the urine of ESCC patients and healthy controls. * *p* < 0.05; ** *p* < 0.01.

SPE-GC-MS was used to detect the concentrations of eight N-nitrosamines in ESCC patients’ urine samples. The results showed that the levels of NDEA, Nmor, and Npyr in the urine samples of 10 ESCC patients were higher than the control group (*p* < 0.05) (Figure 1A,B).

### 3.2. RNA Sequencing Suggests That miR-101-3p Is Down-Regulated in ESCC Tissues

**Figure 2:**
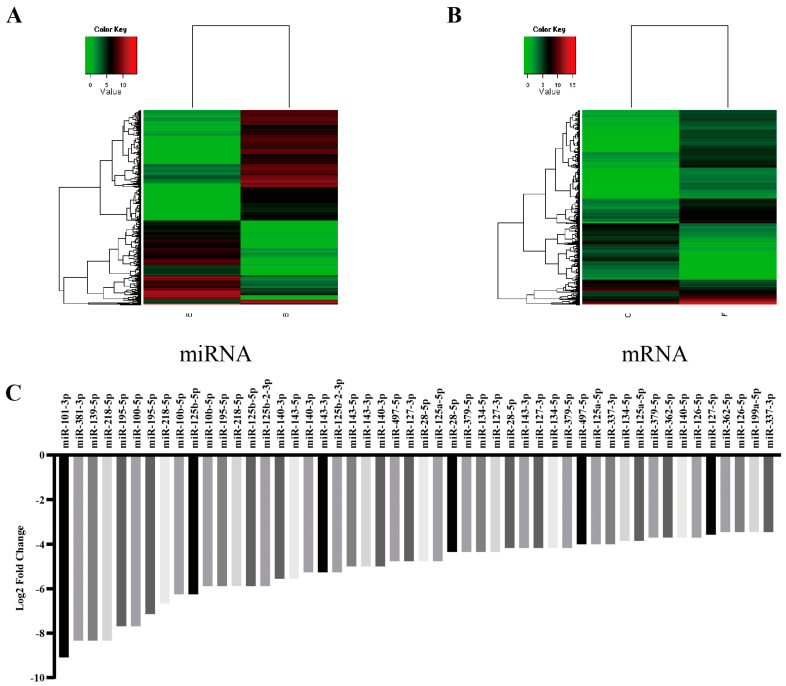
RNA sequencing suggests that miR-101-3p is down-regulated in ESCC tissues. (**A**) Differential miRNAs expression profiles in ESCC and paracancerous tissues (red colour indicates a high level of expression, green colour indicates a low level of expression). (**B**) Differential mRNAs expression profiles in ESCC and paracancerous tissues (red colour indicates a high level of expression, green colour indicates a low level of expression). (**C**) MiRNAs profile with low differentiated expression in ESCC (top 50).

ESCC cancer tissues and their adjacent paracancerous tissues of three patients with new-onset ESCC were screened for miRNA and mRNA differences in the tissues by miRNA and mRNA combination microarrays. A total of 288 miRNAs were differentially expressed in ESCC and adjacent paracancerous tissues; among them, 109 miRNAs were up-regulated in ESCC tissues, and 179 miRNAs were down-regulated. Among them, hsa-miR-101-3p was the most down-regulated (Figure 2).

### 3.3. RT-qPCR Indicates That miR-101-3p Is Down-Regulated in ESCC Tissues and Cells and Can Promote Tumor Growth

**Figure 3:**
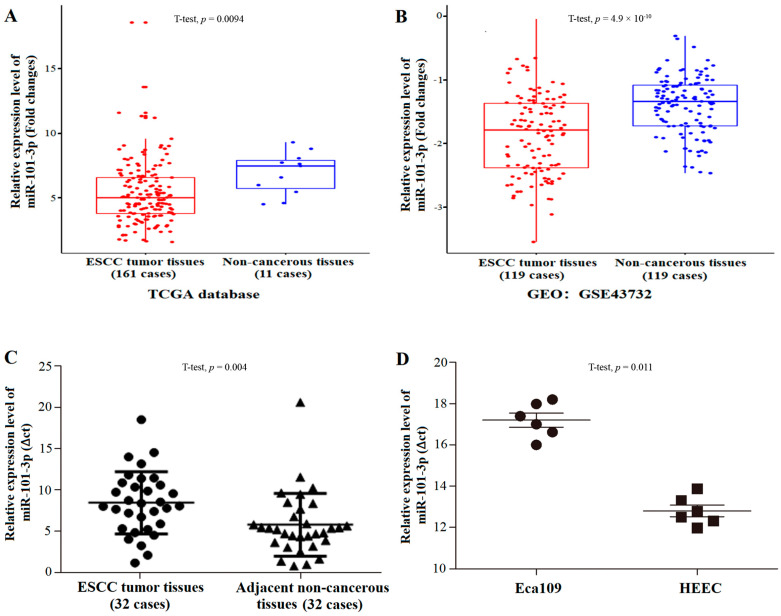
MiR-101-3p is down-regulated in ESCC tissues and cells. (**A**) Expression levels of miR-101-3p in the TCGA database. (**B**) Expression levels of miR-101-3p in the GEO database (GSE43732). (**C**) Relative expression levels of miR-101-3p in 32 pairs of ESCC and paraneoplastic tissues. (**D**) Relative expression levels of miR-101-3p in Eca109 and normal esophageal epithelial cells HEEC.

MiR-101-3p was down-regulated in the tumor tissues of ESCC compared with the non-cancerous esophageal epithelium tissues in the TCGA database (including 161 ESCC tumor tissues and 11 normal esophageal epithelium tissue samples) (Figure 3A). We downloaded the GSE43732 dataset from the GEO and further investigated the actual expression levels of miR-101-3p in the ESCC patient tissues and the non-cancerous esophageal epithelium tissues. The GEO datasets showed a consistent down-regulation of miR-101-3p in ESCC patients’ tumor tissues (Figure 3B).

Subsequently, we chose significant differential expression candidate miR-101-3p to further validate actual expression levels in 32 ESCC patients’ tissue samples. The results showed that miR-101-3p was significantly down-regulated in ESCC tumor tissues (*p* < 0.05) (Figure 3C). The association between miR-101-3p expression levels and the clinicopathological features of the 32 ESCC patients indicated that miR-101-3p was related to tumor size (*p* < 0.05) (Table 3). In addition, miR-101-3p expression in Eca109 and HEEC lines showed that the miR-101-3p was down-regulated in Eca109 cells (*p* < 0.05) (Figure 3D).

### 3.4. Transcription Factors of miR-101-3p Target Genes Are Involved in the Regulation of the Wnt Signaling Pathway

**Figure 4:**
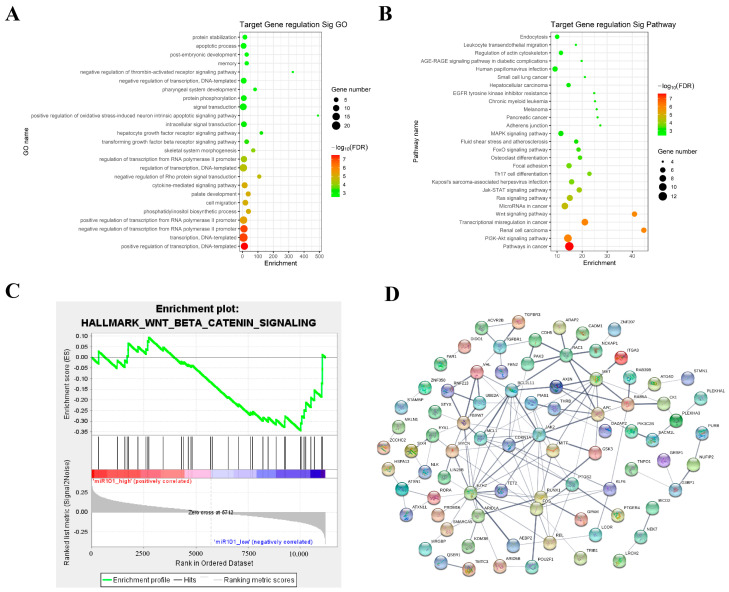
Transcription factors of miR-101-3p target genes are involved in the regulation of the Wnt signaling pathway. (**A**) GO analysis of miR-101-3p target genes. (**B**) MiR-101-3p target gene pathway analysis. (**C**) Gene enrichment analysis. (**D**) Building a PPI network.

We used eight miRNA-target online portal tools to predict target genes, and R 4.4.1 package was used to predict the functions. There were 123 genes considered as potential targets for miR-101-3p, along with 385 potential regulation GO terms and 79 signaling pathways (enrichment score > 1.5, *p* < 0.05). We found that the important GO biological processes related to ESCC are the regulation of transcription (GO:0045893) and cell migration (GO:0016477) (Figure 4A), and these genes enriched cancer-related pathways including the Wnt and PI3K signaling pathways (Figure 4B). Gene set enrichment analysis (GSEA) suggested that the cancer-related signaling pathways include Wnt-β-catenin-signaling, which is the most enriched cancer-related signaling pathway in the miR-101-3p low-expression cases group (*FDR* < 0.05, *p* < 0.05) (Figure 4C).

**Figure 5:**
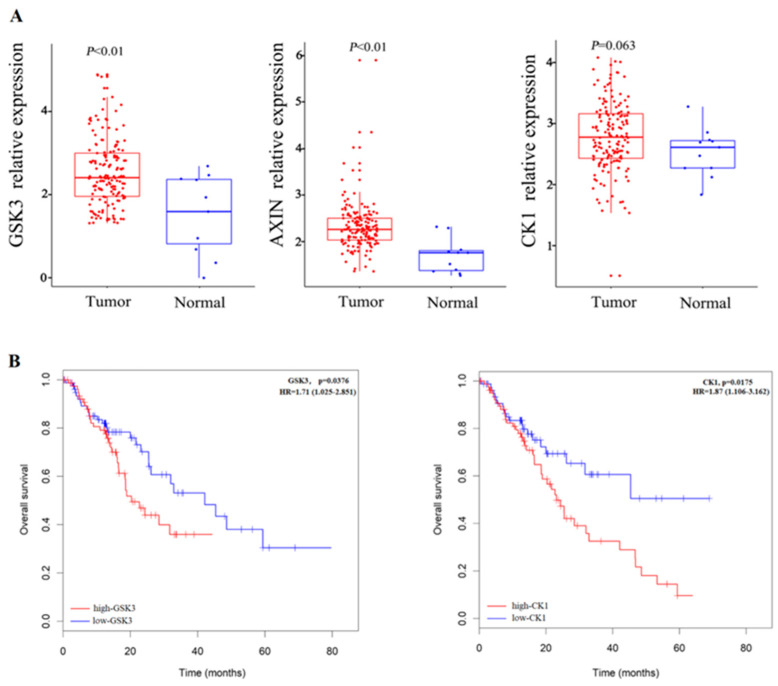
(**A**) Expression of miR-101-3p-related target genes (AXIN, CK1, and GSK3) in the TCGA database in ESCC patients and healthy controls. (**B**) Kaplan-Meier survival analysis validates GSK3 and CK1 with prognostic outcomes in ESCC patients.

We constructed the PPI network and evaluated the potential relationships of these target genes. We found that the transcription factors of miR-101-3p target genes including AXIN, CK1, and GSK3, participated in the regulation of the Wnt signaling pathway, which may indicate that these are miR-101-3p hub target genes that may help us understand the regulation mechanisms in ESCC progression (Figure 4D). Subsequently, we further analyzed the expression of miR-101-3p-related target genes (AXIN, CK1, and GSK3) in ESCC patients and non-cancer cases in the TCGA database. We found that the hub target genes AXIN1 and GSK3 were up-regulated in ESCC patients (Figure 5A). In addition, the overall survival results validated by K-M mapping showed that GSK3 and CK1 were related to the ESCC patients’ prognosis (Figure 5B).

### 3.5. Subchronic Exposure to MNNG Induces Rat Esophagus Damage

**Figure 6:**
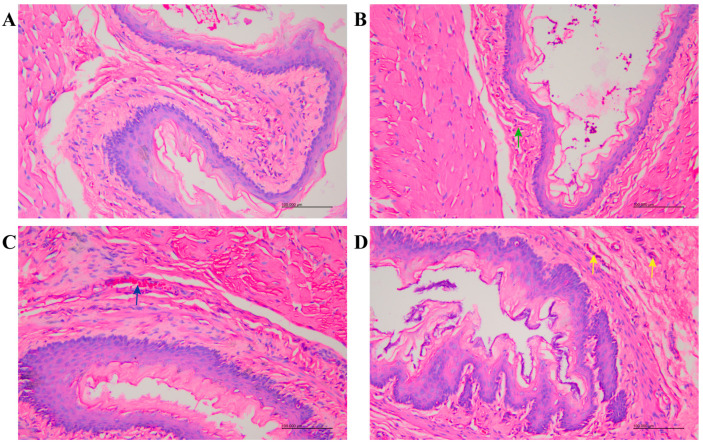
Subchronic exposure to MNNG causes damage to the rat esophagus. (**A**) Control; (**B**) 0.12 mg/kg MNNG-contaminated group, 0.12 mg/kg MNNG group, green arrow shows erosive necrosis of the esophageal mucosa in rats; (**C**) 0.16 mg/kg MNNG group, blue arrow shows submucosal hemorrhage in the rat esophagus; (**D**) 0.20 mg/kg MNNG poisoning group, yellow arrows shows inflammatory cell infiltration in rat esophagus.

Subchronic exposure to MNNG can induce rats’ esophageal mucosal erosion, necrosis, esophageal mucosal keratinization, submucosal hemorrhage, and inflammatory cells in some esophageal tissue sections compared with the control group (Figure 6). The degree of tissue damage increased progressively with higher doses of contamination (Table 4). This indicates that subchronic exposure to MNNG can lead to the destruction of esophageal tissue structure and inflammatory reactions in rats.

### 3.6. Biological Function of miR-101-3p in MNNG-Induced Proliferation of Eca109 Cells

**Figure 7:**
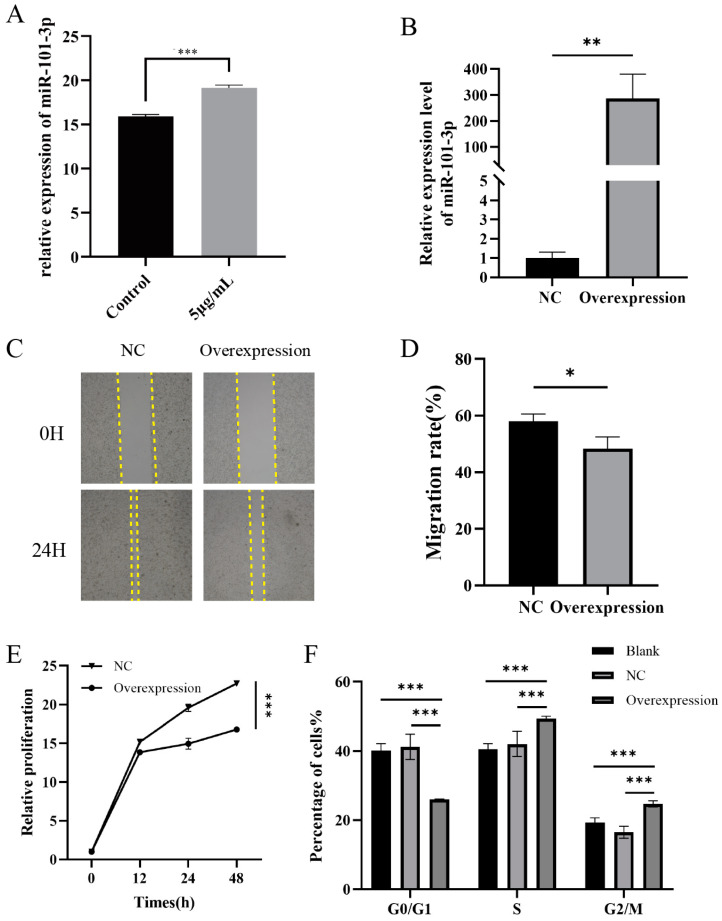
Biological function of miR-101-3p in MNNG-induced proliferation of Eca109 cells. (**A**) RT-qPCR validation of changes in miR-101-3p expression levels after 5 μg/mL MNNG staining of Eca109 cells. (**B**) RT-qPCR validation of miR-101-3p transfection in Eca109 cells. (**C**,**D**) Cell scratch assay. (**E**) CCK8 determines the proliferation level of Eca109 cells after transfection. (**F**) Cell cycle determination by flow cytometry. * *p* < 0.05, ** *p* < 0.01, *** *p* < 0.001.

After the MNNG contamination of Eca109 cells for 48 h, the RT-qPCR assay showed that the expression level of miR-101-3p gene was decreased in the cells of the 5 μg/mL MNNG group (*p* < 0.05) (Figure 7A). By overexpressing miR-101-3p in Eca109 cells for 48 h, we verified its transfection efficiency by RT-qPCR (Figure 7B). A wound healing assay was used to evaluate the Eca109 cells’ migration ability after the miR-101-3p mimic transfection. We found that the wound healing rate was significantly lower in 72 h (*p* < 0.05) (Figure 7C,D). To investigate the effect of the overexpression of miR-101-3p on the proliferation of Eca109 cells, the CCK8 assay was used to determine the changes in the proliferation of Eca109 cells 12 h, 24 h, and 48 h after transfection. The results showed that the Eca109 cell proliferation level was reduced (Figure 7E). The cell cycle results suggested that the G0/G1 phase of the mimic transfection group showed a downward trend, and the G2/M phase showed an upward trend (*p* < 0.05), but there was no significant change in the S phase of the mimic transfection group (Figure 7F).

### 3.7. Regulatory Mechanism of miR-101-3p in MNNG Promoted ESCC

**Figure 8:**
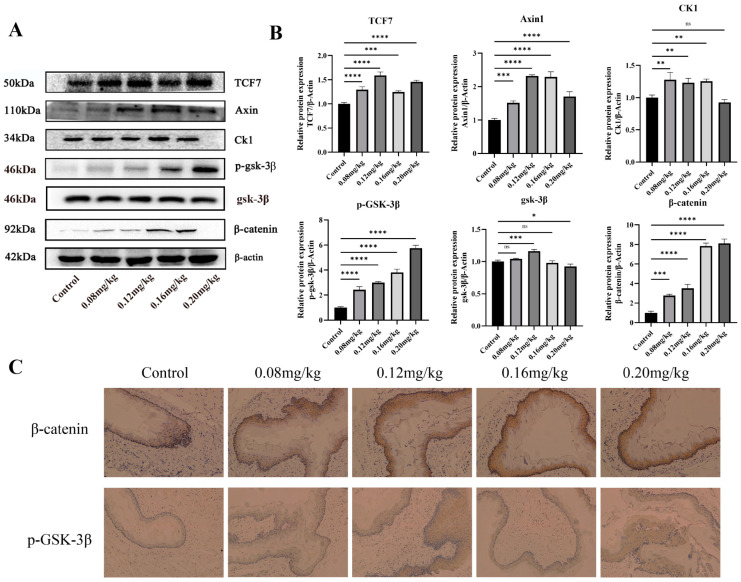
Regulatory mechanism of miR-101-3p in MNNG promoted ESCC. (**A**,**B**) Western blot validation of the Wnt pathway after subchronic MNNG-contaminated rats. (**C**) Immunohistochemical validation of the expression level of key proteins of the Wnt pathway after subchronic intoxication of rats with MNNG. * *p* < 0.05; ** *p* < 0.01; *** *p* < 0.001; **** *p* < 0.0001; ns *p* ≥ 0.05, no significant difference.

The effects of subchronic exposure to MNNG on key proteins of the Wnt signaling pathway in rat esophageal tissues were detected by Western blot. The results showed that the expression levels of CK1, Axin1, and TCF7 proteins in the esophageal tissues of rats in the subchronic exposure group had higher expression (*p* < 0.05) (Figure 8A,B). The immunohistochemical results showed that the β-catenin and p-GSK-3β protein expression were both increased in the esophageal tissues of the subchronically exposed group (Figure 8C).

**Figure 9:**
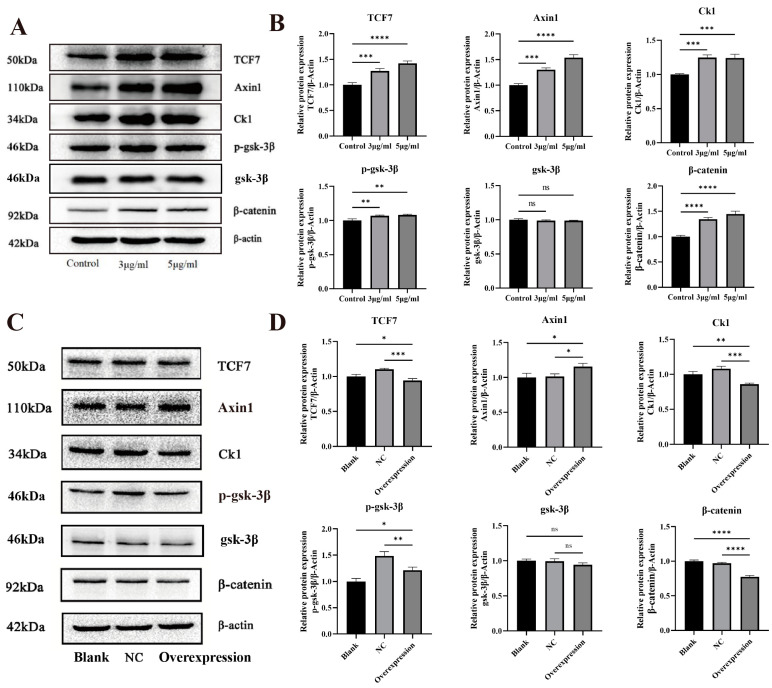
(**A**,**B**) Western blot validation of key proteins of the Wnt pathway after subchronic staining of Eca109 cells with MNNG. (**C**,**D**) Western blot validation of key Wnt pathway proteins after overexpression of miR-101-3p in Eca109 cells. * *p* < 0.05; ** *p* < 0.01; *** *p* < 0.001; **** *p* < 0.0001; ns *p* ≥ 0.05, no significant difference.

After Eca109 cells were exposed to MNNG for 48 h, the key proteins of Wnt signaling pathway were detected. The β-catenin, p-GSK-3β, CK1, Axin1, and TCF7 protein expression levels were significantly increased in Eca109 cells from the MNNG exposed group compared with the control group (Figure 9A,B). The β-catenin, p-GSK-3β, CK1, Axin1, and TCF7 proteins expression levels were reduced after the overexpression of miR-101-3P (*p* < 0.05) (Figure 9C,D).

## 4. Discussion

Esophageal lesions mainly refer to those confirmed by gastroscopy and pathological findings of inflammation and precancerous lesions in the esophagus. The carcinogenesis of esophageal mucosa exhibited obvious bidirectional instability and bidirectional instability in esophageal precancerous lesions (i.e., precancerous lesions in the esophagus may continue to develop into cancer or may return to normal or remain unchanged). In addition, the occurrence of ESCC is the result of complex internal factors and a variety of environmental exposure factors. Therefore, it is crucial to study the genetic susceptibility and environmental risk factors and their interaction in the occurrence of ESCC in the high incidence area to clarify the risk factors, pathogenesis, and formulation of prevention programs for ESCC.

Though surgery, radiotherapy, and chemotherapy have improved the possibilities for recovery, a large proportion of patients with metastatic ESCC still exhibit poor survival rates [10]. Therefore, studies on the etiology, pathogenesis, molecular mechanisms, and biomarkers for the diagnosis and control of ESCC are urgently needed. MNNG is a kind of N-nitroso compound, which has widely existed in the environment as a chemical mutagenic agent and carcinogenic agent and is also a model compound commonly used in the study of the mutagenic mechanism of chemical carcinogens [11]. In daily life, it is mainly ingested through the diet, leading to lesions in the digestive tract [12,13]. By analyzing the epidemiological information of ESCC patients and the urinary N-nitrosamines content of ESCC patients in Wuwei, we found that frequent consumption of preserved food, fried food, and drinking water with excessive N-nitrosamines was closely associated with ESCC.

ESCC-associated miRNAs have been extensively identified and screened in recent years [14]. According to high-throughput detection of tissue microarray chips, we found that the miR-101-3p was the most down-regulated gene in ESCC tumor tissues. MiR-101-3p is affiliated with the miR-101 family and is located on chromosome 1. Studies have also reported that this gene is dysregulated in many diseases, including ovarian cancer, endometrial carcinoma, lung squamous carcinoma, and hepatocellular carcinoma [15,16,17]. MiR-101-3p has also been reported to be down-regulated in tumor tissues of ESCC patients, but its potential regulatory mechanism and exact function have not been elaborated [18]. In our study, we identified the miR-101-3p gene that is abnormally expressed in ESCC by high-throughput sequencing of tissues of patients with high nitrite exposure; then, we analyzed its expression levels in the TCGA database, GSE43732 ESCC dataset, and Eca09 cells. We found that miR-101-3p expression was obviously lower in ESCC tumor tissues in the TCGA database and the GSE43732 dataset. This result was consistent with the 32 newly diagnosed ESCC population samples, and the ESCC cell line was RT-qPCR verified. Meanwhile, this study concludes that the miR-101-3p in ESCC may also have diagnostic value.

The present study demonstrated that down-regulated miR-101-3p expression in ESCC tissues might be associated with the development of ESCC [19]. To find the potential function and regulation mechanisms of miR-101-3p, we conducted a GSEA of ESCC data from the TCGA database and R 4.4.1. The GO analysis and pathway results show that the abnormal regulation of the Wnt pathway plays an important role in the process of ESCC. Compared with the function reports of previous miRNA target genes, we used more predictive software to narrow the range of the target genes to make our results more reliable [20,21]. In our study, we predicted the relationship between the target gene and miR-101-3p expression, further analyzed its function, and then further constructed a PPI network to understand deeper functions.

PPI is the process of forming complexes between proteins [22]. Three genes (AXIN1, CK1, and GSK3) are regarded as the central genes in ESCC. According to existing research reports, the Wnt pathway is related to the progression of many cancers and is crucial for early intestinal morphogenesis and intestinal phenotype maintenance [23,24,25]. By signaling Wnt glycoprotein secretion or mitosis and the nuclear translocation of dephosphorylated β-catenin, the typical Wnt/β-catenin signal is activated [26]. Wnt signaling is not present, and β-catenin is separated from the destruction complex [27]. In addition, the key protein of the Wnt pathway, AXIN1, acts directly on β-catenin, GSK3, and APC and is directly related to the formation of the β-catenin destruction complex [28]. Classic Wnt signaling is also related to tumor development in leukemia subtypes and is essential for leukemia-initiating cells [29]. Wnt signaling is activated in more than one-half of breast cancer patients and can reduce overall survival [30]. Many studies have verified that the Wnt pathway can regulate cell cycle progression, cell proliferation and survival, and cell apoptosis [31,32,33,34]. These results suggest that miR-101-3p dysregulation may play a crucial role in the ESCC process; therefore, we chose the Wnt pathway for further study. It was shown that MNNG induced human gastric mucosal epithelial GES-1 cells, and it was found to activate the Wnt/β-catenin pathway and up-regulate key proteins [35]. Chronic exposure to MNNG increases expression levels of key proteins in the Wnt/β-catenin pathway in rat gastric tissues, such as Wnt2β, PCNA, and β-catenin [36,37]. Our study found that subchronic exposure to MNNG caused the esophagus of rats to develop esophageal mucosal erosive necrosis, submucosal hemorrhage, and inflammatory cell infiltration.

In the present study, we overexpressed miR-101-3p in Eca109 cells and found that the proliferative and migratory capacities of the cells were significantly reduced; then, we selected the Wnt signaling pathway as a relevant signaling pathway in ESCC and performed Western blot analysis to confirm whether there is an association between subchronic exposure to MNNG and miR-101-3p and the key genes of the Wnt pathway. We found that subchronic exposure to MNNG was able to activate the Wnt/β-catenin pathway, leading to a significant increase in the expression levels of key proteins in the pathway. Through the overexpression of miR-101-3p, we observed the opposite result. These findings suggest that the Wnt signaling pathway is activated during the precancerous stage of ESCC induced by MNNG. Furthermore, the expression levels of key proteins involved in this pathway, including TCF7, AXIN1, CK1, GSK3, and β-catenin, were increased. Conversely, the overexpression of miR-101-3p resulted in the inhibition of the Wnt signaling pathway and a corresponding decrease in the expression levels of these key proteins, suggesting that the expression levels of miR-101-3p may be one of the protective factors during ESCC progression.

In this study, we also collected epidemiological data and urine samples from patients with ESCC, which were analyzed. It was found that the patients often drank water with high N-nitrosamines and consumed preserved food, fast food, and convenience food. Subsequently, we used high-throughput chip sequencing and found that miR-101-3p was the most down-regulated gene in the ESCC cancer tissues. We also used TCGA and GEO databases, patient tissues, and cell lines to analyze miR-101-3p expression. Mutual verification ensures the accuracy and reliability of our results. We performed GESA, GO, and Pathway analyses and built the PPI network to predict the target genes of miR-101-3p. After the over-expression of miR-101-3p in Eca109 cells, the potential biological function was verified, and the mechanism was studied. We also validated the potential molecular mechanisms that cause damage to the esophagus after subchronic exposure to MNNG in rats. However, there are limitations to this study. Future studies should verify more datasets, and the effects of the over-expression of miR-101-3p on other ESCC cell lines should be studied.

## 5. Conclusions

Our study showed that MNNG was able to cause esophageal epithelial damage. miR-101-3p was the most down-regulated gene in ESCC and has diagnostic and prognosis value. Meanwhile, miR-101-3p may also affect the growth of ESCC by adjusting the Wnt signaling pathway and may function as an antioncogene and play an important role in ESCC tumorigenesis. Most importantly, we expect that miR-101-3p can be a prospective biomarker of ESCC in future diagnostic classifications and prognoses.

## Figures and Tables

**Table 1:** miR-101-3p and U6 primer sequences.

Gene	Primer	Sequence
miR-101-3p	Forward primer	GCGCGCGTACAGTACTGTGATA
	Reverse primer	AGTGCAGGGTCCGAGGTATT
	PT primer	GTCGTATCCAGTGCAGGGTCCGAGGTATTCGCACTGGA TACGACTTCAGT
U6	Forward primer	CTCGCTTCGGCAGCACA
	Reverse primer	AACGCTTCACGAATTTGCGT

**Table 2:** Analysis of the correlation between lifestyle behaviors of 60 esophageal cancer patients and 180 control subjects.

Variables	N	ESCC Patients (%)	Normal (%)	*p*
Smoking				0.0691
≥7 days/week	59	20 (33.90%)	39 (66.10%)	
<7 days/week	181	40 (22.10%)	141 (77.90%)	
Alcohol				0.1932
≥3 days/week	72	22 (30.56%)	50 (69.44%)	
<3 days/week	168	38 (22.62%)	130 (77.38%)	
Tea				0.0025 *
≥5 days/week	71	27 (38.03%)	44 (61.97%)	
<5 days/week	169	33 (19.53%)	136 (80.47%)	
Source of drinking water				0.0111 *
Tap water	57	7 (12.28%)	50 (87.72%)	
Well, lake or river water	183	53 (28.96%)	130 (71.04%)	
Preserved foods				<0.0001 *
≥4 days/week	111	44 (39.64%)	67 (60.36%)	
1–3 days/week	110	10 (9.09%)	100 (90.91%)	
Rarely	19	6 (31.58%)	13 (68.42%)	
Fried foods				0.6373
≥4 days/week	158	41 (25.95%)	117 (74.05%)	
1–3 days/week	82	19 (23.17%)	63 (76.83%)	
Fast food and convenience food				0.0514
≥4 days/week	85	15 (17.65%)	70 (82.35%)	
1–3 days/week	155	45 (29,03%)	110 (70.97%)	
Total	240	60	180	

* *p* < 0.05, suggesting that the difference is statistically significant.

**Table 3:** The relationship between miR-101-3p and 32 ESCC clinicopathological parameters.

Variables	Cases, *n* (%)	Low, *n*	High, *n*	*p*
Gender				0.121
Male	27 (84.38)	21	6	
Female	5 (15.62)	2	3	
Age				0.444
≤60	17 (53.13)	11	6	
>60	15 (46.87)	12	3	
TNM stage				0.535
I + II	9 (28.13)	6	3	
III + IV	23 (71.87)	17	6	
Lymph node status				0.654
Metastasis	8 (25)	5	3	
NO	24 (37)	18	6	
Tumor area (cm^2^)				0.002 *
≥5	24 (75)	21	3	
<5	8 (23)	2	6	

* *p* < 0.05, suggesting that the difference is statistically significant.

**Table 4:** Histopathological damage scores.

Groups	Score	F	*p*
control	1.167 ± 0.4082	25.48	<0.0001 *
0.12 mg/kg	1.833 ± 0.4082		
0.16 mg/kg	2.500 ± 0.5477		
0.20 mg/kg	3.500 ± 0.5477		

* *p* < 0.05, suggesting that the difference is statistically significant.

## Data Availability

Data will be made available on request.
